# Buccal rotation for wholly impacted maxillary third molar extraction

**DOI:** 10.1186/s13005-023-00348-3

**Published:** 2023-01-30

**Authors:** Zhou-Xi Ye, Wen-Hao Qian, Yu-Bo Wu, Chi Yang

**Affiliations:** 1 Shanghai Xuhui District Dental Disease Prevention and Control Institute, No. 500, Fenlin Rd, Shanghai, People’s Republic of China; 2 Shanghai Ninth People’s Hospital, Shanghai Jiao Tong University School of Medicine, Shanghai, China

**Keywords:** Impacted maxillary third molar, Tooth extraction, Impacted position, Angulation, Surgical simulation

## Abstract

**Background:**

Extracting wholly impacted maxillary 3rd molars faces difficulty due to the narrow surgical field, adjacent teeth resistances and risk of oroantral communication. This study is designed to introduce and evaluate the applicability of a novel method-buccal rotation to extract maxillary 3rd molars.

**Materials and methods:**

In this cohort study, from October 1st 2020 to September 30th 2021, 72 wholly impacted maxillary 3rd molars were included. Based on the crowns with coronal 1/3, middle 1/3, apical 1/3 of the adjacent teeth roots, teeth were classified into position I, II, III. Based on the angles < 30°, ≥ 30°but < 60°, ≥ 60° to the adjacent teeth, teeth were classified into angulation A, B, C. Traditional method and novel method-buccal rotation were applied based on the surgical simulations. Surgical results were recorded. To analyze the data, Chi-square test was applied.

**Results:**

82.00% of teeth in position I and 50.00% in position II were designed to use traditional method, 83.33% in position III were using the novel method (*p* < 0.05). 81.25% of teeth in angulation A and 52.63% in angulation B were designed to use traditional method, 80.00% in angulation C were using the novel method (*p* < 0.05). Four cases got temporary complications.

**Conclusion:**

Buccal rotation was applicable to extract the deep impacted maxillary third molars with large angles towards the adjacent teeth.

## Background

The extractions of wholly impacted maxillary third(3rd) molars are challenges to oral surgeons due to the following reasons: (1) limited surgical field; (2) large adjacent tooth resistance [1]; (3) the proximity to maxillary sinus [2–5]. Therefore, any unplanned surgery would easily cause excessive surgical trauma, increased adjacent teeth movements [1] and oroantral communications [2]. To avoid the adjacent tooth resistance and to create a wider surgical field, a novel method is required to be introduced. Buccal rotation of the teeth could decrease the distal bone loss of the adjacent teeth and might be an ideal novel method to extract the wholly impacted maxillary 3rd molars.

According to the literary, although there were some studies mentioning surgical approaches for difficult impacted maxillary 3rd molars [6–10] (mostly described in case reports), However, according to our knowledge, none of them had introduced the method buccal rotation in detail. Three problems are to be answered as follows: (1) for the wholly impacted maxillary 3rd molars, is there a comprehensive evaluation with the help of three-dimensional (3D) reconstruction? (2) in the novel method buccal rotation, what is the key technique? (3) what kind of teeth are the most recommended to use the novel method? The study is an observational cohort study, which aims at presenting the surgical design, procedure, and the novel surgical method, as well as its clinical value.

## Methods and materials

### Patients

This is an observational cohort study. The study followed the Declaration of Helsinki on medical protocol and ethics, and the regional Ethical Review Board of Shanghai Xuhui District Dental Disease Prevention and Control Institute approved the study (XYD AF/SC-08/01.0). All patients were informed about surgical purpose, procedure, recovery period, and possible complications. All patients signed a consent form. From October 2020 to September 2021, a consecutive group of 725 patients with 953 maxillary 3rd molars were reviewed. The inclusion criteria were wholly impacted (bony impacted) 3rd molars. In total, 58 patients with 72 maxillary 3rd molars were included. Every patient was gained a detailed medical and dental history, both orthopantomography (OPG) and cone-beam computed tomography (CBCT) of the surgical site was taken in every patient.

### Tooth position and angulation

**Fig. 1:**
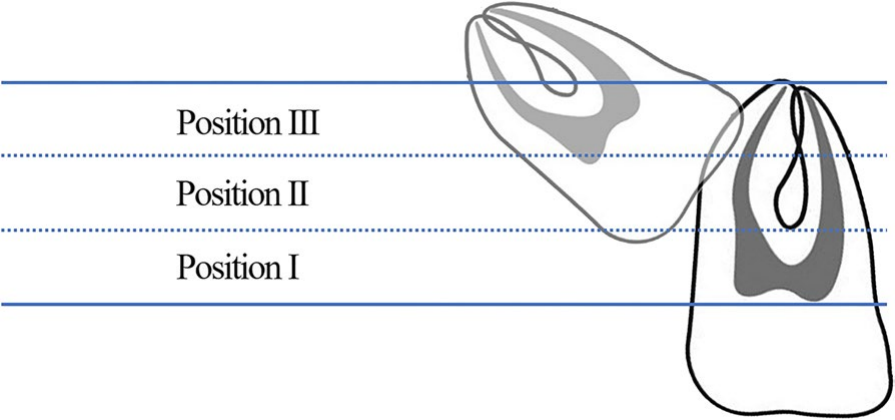
Tooth position classification based on the relative impaction depth

According to the impaction depth, the included maxillary 3rd molars were classified into three classes (Fig. 1):

- Position I: The lowest portion of the impacted crown was on a level on/below the coronal 1/3 of the second (2nd) molar’s root.
- Position II: The lowest portion of the impacted crown was on a level with the middle 1/3 of the 2nd molar’s root.
- Position III: The highest portion of the impacted crown was on a level on/above the apical 1/3 of the 2nd molar’s root.

**Fig. 2:**
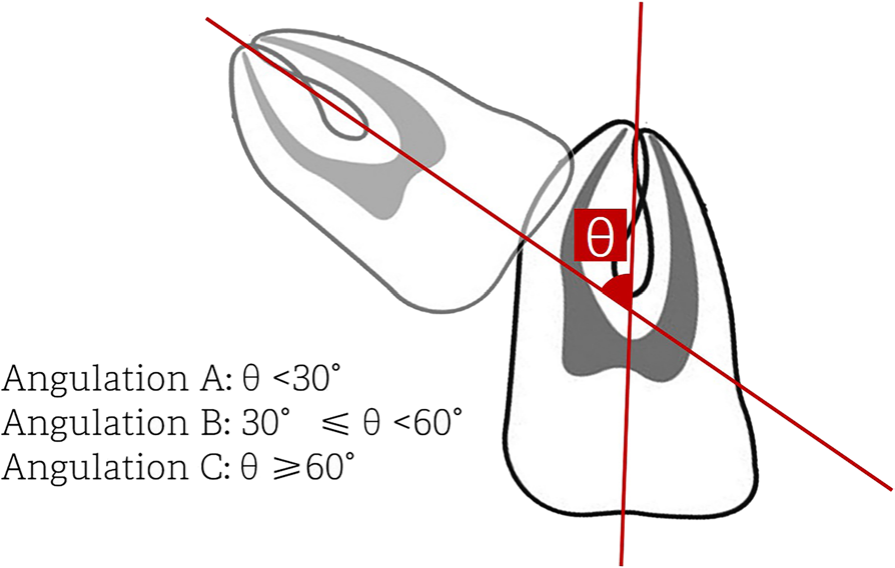
Tooth angulation classification based on the angle between the long axes of the maxillary third molar and the maxillary second molar

According to the angle between the long axes of the maxillary 3rd molar and the maxillary second molar, the included maxillary 3rd molars were classified into three classes (Fig. 2):

- Angulation A: The angle between the long axes was less than 30°.
- Angulation B: The angle between the long axes was over 30°(30°included), but less than 60°.
- Angulation C: The angle between the long axes was over 60°(60°included).

### Surgical design and 3D surgical simulation

**Fig. 3:**
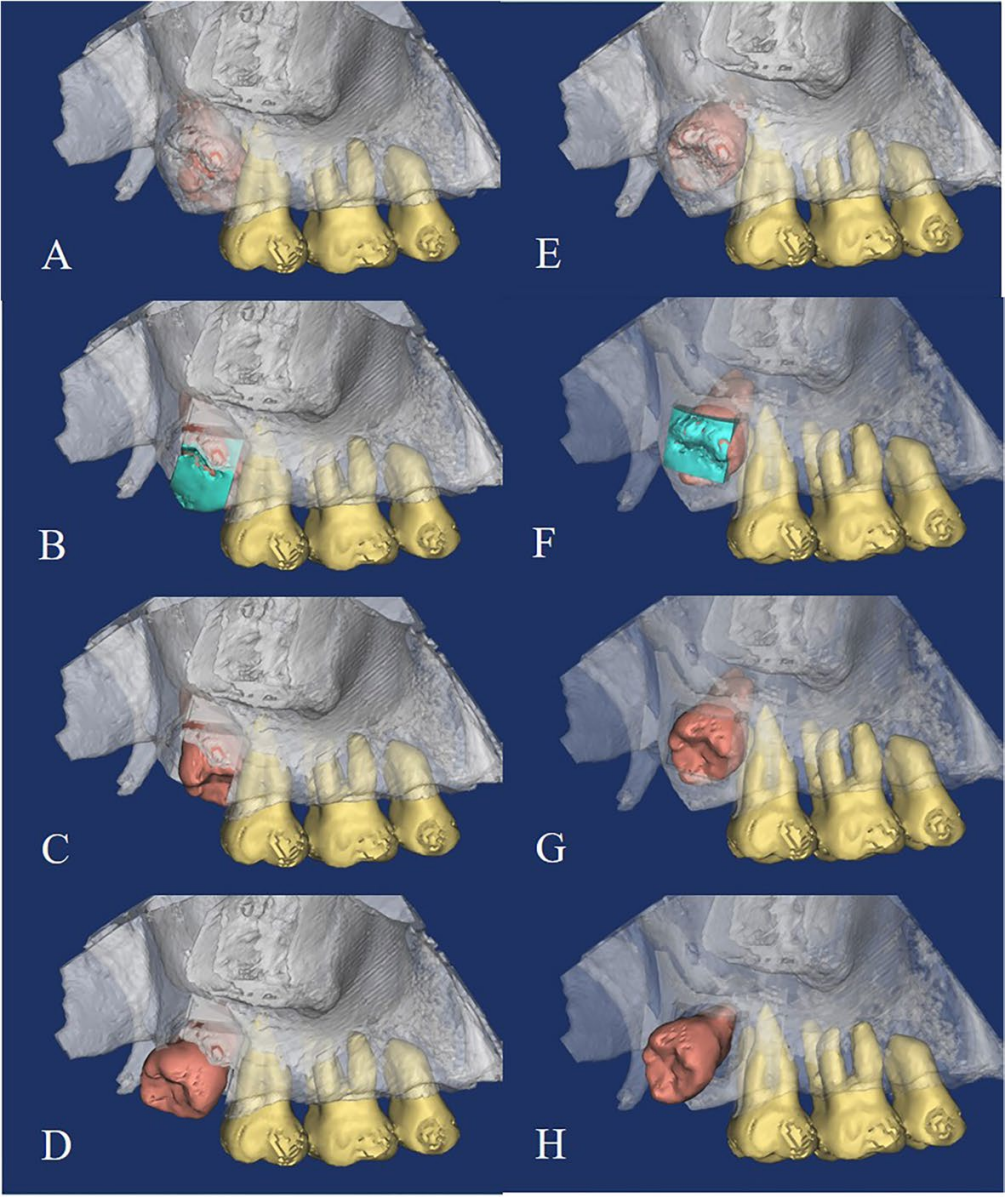
The surgical design and simulation based on CBCT reconstruction model. **A**, **B**, **C**, **D** showed the surgical design of the traditional method occlusal rotation; **E**, **F**, **G**, **H** showed the surgical design of the novel method buccal rotation

The surgical design and simulation in each case includes the following steps: (1) to make the 3D reconstruction, CBCT data was transferred into mimics 21.0 (Materialize Co, Leuven, Belgium). (2) teeth, alveolar bone was segmented out. (3) on the 3D model, simulating osteotomy and tooth rotation (Fig. 3).

All surgical simulations could be concluded into two types based on the surgical design as follows:

(1) traditional extraction method: to rotate the impacted tooth distally through the occlusal approach (Fig. 3A,B,C,D).

(2) novel extraction method: to rotate the impacted tooth in the distobuccal direction through the buccal approach (Fig. 3E,F,G,H).

### Surgical procedure

Under local anesthesia with 4% Articaine, all patients were operated by one surgeon (Ye ZX) with 10-year clinical experience. The extraction procedures were in accordance with the design. A full thickness flap elevated and a dental handpiece (Sirona, Germany) was implemented for bone removal. In the novel extraction, the bone resistance was carefully released with the handpiece. Then the crown was rotated distobuccally with the elevator located on its mesial side. The sockets were closed by 4–0 absorbable silk sutures (Fuxin, China) after the surgery.

### Outcome assessment

In this study, outcome variables were success rate, operating time, recovery, and the incidence of major complications. Patients were recalled 1 week, 1 month, and 3 months after the extraction.

Visual analog scale (VAS) method was used to evaluate pain level. Swelling was measured with a standard calipers from the mesial aspect of the maxillary first molar crown to the tangent of the cheek skin. Major severe complications include maxillary 2nd molar injury and oroantral communication. The clinical features of oroantral communication were the unhealed wounds after operation and the patients felt leak air during cheek blowing.

In this study, data was collected in a spreadsheet (Excel; Microsoft Inc, Redmond, WA) and analyzed with SAS 12.0 (SAS institute, USA). Chi-square test and Fisher’s Exact Test were used to analyze the data. A value of *p* ≤ 0.05 was considered statistically significant.

## Results

There were 72 teeth in the 58 patients included in this study, with ages ranged from 19 to 42 years (average, 27 years).Of these, 34 were female (41 teeth), and 24 were male (31 teeth).

There were 50 teeth were in position I. Among them, 41 of them designed to be extracted using the occlusal rotation method, and 9 designed using buccal rotation; 16 teeth were in position II, with 8 designed using the occlusal rotation method, and 8 designed using buccal rotation; 6 teeth were in position III, with 5 designed using buccal rotation (*p* < 0.05), as presented in Table 1.

**Table 1:** Cases involved major complications

Classification	Method		*p*
	Occlusal rotation (traditional method)	Buccal rotation (novel method)	
position			
I	41	9	
II	8	8	
III	1	5	< 0.05
Angulation			
A	39	9	
B	10	9	
C	1	4	< 0.05

In total,48 teeth were classified into Angulation A, with 39 of them designed to be removed using the occlusal rotation method, and 9 designed using crown buccal rotation; 19 teeth were classified into Angulation B, with 10 of them designed to be removed using the occlusal rotation method, and 9 designed to rotate buccally; 5 teeth were classified into Angulation C, with 4 designed using buccal rotation (*p* < 0.05), as presented in Table 1.

The success rate of extractions in this study was 100%. The average operating time was 13.5 min. The VAS score and swelling measurement in patients were 0.69 and 0.18 cm one week after surgery.

One case in position II and angulation C using occlusal rotation method was found temporarily increased maxillary 2nd molar movement, and recovered in 1 month after surgery. One case in position III and angulation C using the buccal rotation method was found temporarily increased maxillary 2nd molar movement and recovered in 3 months after surgery. One case in position II and one case in position III were found oroantral communications and recovered in 1 month after extraction. The flap surgeries were applied at one week after the extraction in cases with oroantral communication. The main steps of the flap surgery were local gingiva flap release under local anesthesia, flap traction to cover the wound, and suture. The other major complications was not found in this study (Table 2).

**Table 2:** Position and angulation classification of maxillary 3rd molars and their extraction methods. *p ≤ 0.05 is considered statistically significant

Patient list	gender	Position classification	Angulation classification	Method	Major complication	Recovery time
1	male	II	C	Occlusal rotation	Maxillary 2^nd^ molar injury	1 month
2	female	III	C	Buccal rotation	Maxillary 2^nd^ molar injury	3 months
3	male	II	B	Occlusal rotation	Oroantral communication	1 months
4	female	III	B	Occlusal rotation	oroantral communication	1 months

## Discussion

The wholly impacted maxillary 3rd molars are difficult to extract, due to the limited surgical view and the risk of increased adjacent tooth movements and oroantral communications [1–5]. With the soft tissue surrounded and the adjacent teeth erupted distally, the surgical field is usually narrow and should be carefully widened with small retracts. The risk of adjacent tooth injury is related to two factors impaction depth and the angle towards the adjacent tooth. The deeper the tooth impacted, the larger amount of bone is needed to be removed. Thus, it leads to the loss of distal support to the adjacent tooth^1^. The traditional method uses the occlusal approach, which means the distal bone support would be removed to the level of the impacted crown. Therefore, it calls for a novel method to remove bone in another approach, especially when deep impacted teeth are needed to be extracted. The bigger the angle between the axes of the impacted tooth and the adjacent tooth is, the larger crown resistance is existed during extraction [11]. Although the remove of the distal bone of the impacted tooth crown is relatively not hard due to the low bone density of the maxilla, the buccal approach could avoid almost all crown resistance, which reduces the adjacent tooth injury risk. The oroantral communication is associated with the proximity of the tooth and maxillary sinus. In literary, the impacted maxillary 3rd molars could be divided into several types based on the orthopantomography (OPG) image features between the tooth root and the sinus bottom white line [12, 13]. However, it could only indicate the possible relationship of the tooth and sinus. To show the detailed relationship, CBCT should be taken [14]. In this study, the focus is not located on this topic, but the surgeons could recognize the oroantral communication risks because all included cases had taken CBCT examinations. The oroantral communication risk is related to the impaction depth and the surgical approach. The proximity is closer between the teeth and sinuses when the teeth impact in deeper positions. For the teeth in close proximity to the sinus, the extraction approach could make passage into the oral environment and the sinus [3]. And without a tight and perfect closure and adequate postoperative care, the oroantral communication could be occurred after surgery. Because the surgical incision is on or near the alveolar crest, the occlusal approach is easier to make this communication due to the close location between the suture and the tooth dislocation passage [15, 16]. In the buccal approach, the tooth dislocation passage is on the buccal side, which is away from the suture. Thus, this novel approach could help decrease this risk. Considering all risk factors above, a novel method to extract impacted maxillary third molars in a buccal approach was introduced and implemented in this study.

For maxillary 3rd molars, OPG could be used to evaluate the surgical difficulty. But for the impacted ones, CBCT could show more details, which valued in guiding the surgery [17–19]. Besides, the CBCT data could be transferred for surgical simulation, which has been a hot topic in bone surgery in recent years [20, 21]. In our study, it has been proven valid in evaluation of the 3D reconstruction of CBCT and surgical simulation. The limitations of this computer technique are time-consuming, but it will benefit in pre-surgery patient communication and helping surgical design. With artificial intelligence widely applied in surgical design, it might help to lighten the workload of surgeons in the future.

The surgical procedures of the novel method include the following steps:(1)make incisions near the distal alveolar crest of the maxillary second molar; (2)remove the buccal bone to expose the crown of the impacted tooth based on the surgical design; (3)rotate the impacted crown disto-buccally and extract the whole tooth in buccal approach; (4) embolize gelfoam and make tight sutures. The key step of the surgery was the crown rotation with adequate bone removal. The bone removal side was chosen according to the impaction depth shown in CBCT and its reconstruction. A guiding the pad made before the surgery would contribute to make a more precise location of bone removal [22]. However, it will increase the presurgical work and the surgical cost. It could be a research direction in the future when a low-cost and fast manufacturing guiding pad is introduced.

In this study, deeper impacted maxillary 3rd molars were more likely to use the novel extraction method. It was shown in the study that 82.00% of teeth in position I and 50.00% of teeth in position II were designed using the traditional method, while 83.33% teeth in position III were designed using the novel method (*p* < 0.05).The possible explanations are listed as follows:(1) there was larger bone resistance of the occlusal side of the impacted crown than the buccal side in the deep impacted tooth; (2) the deeper impacted tooth usually has a closer relationship with the sinus [12], the novel method could decrease the oroantral communication risk as it has been discussed. This study found the teeth which had larger angles with the adjacent teeth were more likely to be extracted using the novel method. The teeth impacted with large angles to the adjacent teeth had large resistances from the adjacent teeth [11]. Therefore, it could be suggested that the novel method was applicable and could reduce even avoid the crown resistance of the adjacent teeth.

The complications happened in this study were mild and acceptable. And all patients recovered no longer than 3 months. In total, 4 cases had complications. Among them, two cases suffered with increased adjacent tooth movements, while two cases got oroantral communication. They were all deep impacted (positions II and III) and had large impacted angles towards the adjacent teeth (angulations B and C). Three of them were extracted using the traditional method, while one was removed using the novel method, indicating that the novel method might help to reduce the risk of complications. The limitation of this study is the observational character. A multi-center with a large sample trial is needed to lead to more meaningful conclusions.

## Data Availability

All data generated or analyzed during this study are included in this published article.

## Abbreviations

3D: Three-dimensional
CBCT: Cone-beam computed tomography
OPG: Orthopantomography
VAS: Visual analog scale
